# Garlic-Derived Allicin Attenuates Parkinson’s Disease via PKA/p-CREB/BDNF/DAT Pathway Activation and Apoptotic Inhibition

**DOI:** 10.3390/molecules30153265

**Published:** 2025-08-04

**Authors:** Wanchen Zeng, Yingkai Wang, Yang Liu, Xiaomin Liu, Zhongquan Qi

**Affiliations:** School of Medicine, Guangxi University, Nanning 530004, China

**Keywords:** allicin, Parkinson’s disease, network pharmacology, apoptosis

## Abstract

Allicin (ALC), a naturally occurring organosulfur compound derived from garlic (*Allium sativum*), exhibits potential neuroprotective properties. Parkinson’s disease (PD) is a progressive neurodegenerative disease characterized by degeneration of dopaminergic neurons and motor dysfunction. This study utilized bioinformatics and network pharmacology methods to predict the anti-PD mechanism of ALC and established in vivo and in vitro PD models using 6-hydroxydopamine (6-OHDA) for experimental verification. Network pharmacological analysis indicates that apoptosis regulation and the PKA/p-CREB/BDNF signaling pathway are closely related to the anti-PD effect of ALC, and protein kinase A (PKA) and dopamine transporter (DAT) are key molecular targets. The experimental results show that ALC administration can alleviate the cytotoxicity of SH-SY5Y induced by 6-OHDA and simultaneously improve the motor dysfunction and dopaminergic neuron loss in PD mice. In addition, ALC can also activate the PKA/p-CREB/BDNF signaling pathway and increase the DAT level in brain tissue, regulate the expression of BAX and Bcl-2, and reduce neuronal apoptosis. These results indicate that ALC can exert anti-PD effects by up-regulating the PKA/p-CREB/BDNF/DAT signaling pathway and inhibiting neuronal apoptosis, providing theoretical support for the application of ALC in PD.

## 1. Introduction

Parkinson’s disease (PD), as a common neurodegenerative disorder, has garnered significant attention due to its progressively worsening motor impairments and physiological dysfunctions. According to statistics from the *Lancet Healthy Longev*, the global prevalence of PD has been steadily increasing, with an average incidence rate of 3.81 cases per 1000 individuals from 2010 to 2023 [1]. The death of dopaminergic neurons has been identified as a core pathological feature of PD, resulting in motor symptoms such as tremors, rigidity, bradykinesia, and postural instability, along with non-motor symptoms, severely impairing the patients’ quality of life [2]. Currently, the treatment of PD primarily focuses on symptom management rather than a cure. Treatment approaches include pharmacological therapy [3], surgical intervention, and non-pharmacological interventions. Commonly used medications for treating PD, such as levodopa, can produce various side effects after prolonged use, making the search for new therapeutic agents imperative. The pathogenesis of PD remains incompletely understood; however, an increasing body of evidence suggests that multiple factors, including apoptosis, oxidative stress, and protein aggregation, play significant roles in the development of this disease [4,5].

Apoptosis is a form of programmed cell death that can occur via receptor-mediated and mitochondrial-mediated pathways. In PD, the mitochondrial-mediated apoptosis pathway is considered a principal mechanism underlying the death of dopaminergic neurons, regulated by pro-apoptotic and anti-apoptotic members of the Bcl-2 protein family [6,7]. It has been confirmed that the expression level of BAX is elevated and the level of Bcl-2 is decreased in the brain tissue or peripheral lymphocytes of patients with PD [8,9,10]. Research has demonstrated that the PKA/p-CREB/BDNF pathway plays a crucial role in neuroprotection and cell survival. Protein kinase A (PKA) promotes the expression of brain-derived neurotrophic factor (BDNF) by phosphorylating cAMP response element-binding protein (CREB). Following its binding to the TrkB receptor, BDNF can further promote the phosphorylation of CREB, thereby enhancing BDNF transcription [11]. Furthermore, activated CREB can bind to the corresponding sequences in the *TH* gene promoter, increasing *TH* transcription [12]. Tyrosine hydroxylase, encoded by the *TH* gene, is the rate-limiting enzyme in dopamine synthesis and plays a critical role in PD. Numerous studies indicate that activating the PKA/p-CREB/BDNF pathway can inhibit the apoptosis of dopaminergic neurons, thereby improving behavioral deficits associated with PD [13,14].

*Allium sativum* (garlic) is not only a widely used seasoning but is also considered to have medicinal value in traditional medicine, being utilized for the treatment of various diseases, including hypertension, hyperlipidemia, infections, and cancer [15,16]. Moreover, the neuroprotective effect of garlic in PD has been validated by multiple studies [17,18]. Allicin (ALC) is a bioactive compound extracted from crushed garlic, which readily crosses the blood-brain barrier and exhibits significant antioxidant, anti-inflammatory, and neuroprotective effects [19,20,21]. Research indicates that ALC can inhibit the apoptosis of 6-hydroxydopamine (6-OHDA)-induced PC12 cells by alleviating mitochondrial dysfunction [22]. However, a systematic and in-depth exploration of the specific mechanisms by which ALC operates in the treatment of PD is currently lacking. In this context, utilizing bioinformatics and network pharmacology methodologies, we have predicted the key targets and signaling pathways of ALC against PD, discovering that its mechanisms of action may be associated with the inhibition of apoptosis and the upregulation of the PKA/p-CREB/BDNF pathway. By thoroughly investigating the mechanisms of action of ALC, this study will provide scientific evidence for its pharmacological properties, aiming to serve as a valuable reference for research in this field.

## 2. Results

### 2.1. Identification of ALC Targets and PD Hub Genes

ALC targets were retrieved from SwissTargetPrediction, PubChem, SymMap, PharMapper, CTD, and STITCH databases, resulting in a total of 278 unique targets.

**Figure 1:**
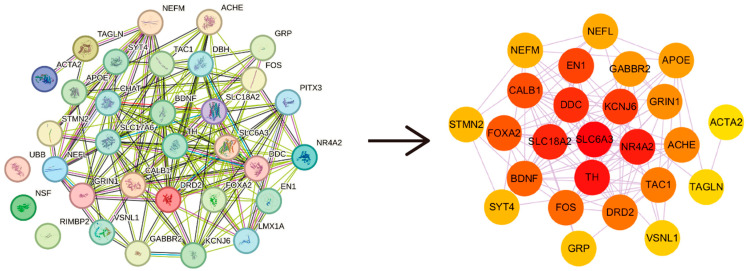
Identification of PD hub genes.

Differentially expressed genes were identified from five PD-related datasets. The differentially expressed genes were input into STRING to generate a protein–protein interaction (PPI) network. The top ten genes with the highest Maximum Clique Centrality (MCC) scores were identified as hub genes using the CytoHubba plugin in Cytoscape. After removing duplicates, a total of 28 hub genes were identified (Figure 1). The top ten hub genes are as follows: *SLC6A3*, *TH*, *SLC18A2*, *NR4A2*, *DDC*, *KCNJ6*, *EN1*, *FOXA2*, *BDNF*, and *DRD2*.

### 2.2. Mechanism Exploration of ALC Anti-PD

Kyoto Encyclopedia of Genes and Genomes (KEGG) and Gene Ontology (GO) analyses were performed on the PD hub genes and ALC targets to investigate their pathogenesis and regulatory mechanisms.

**Figure 2:**
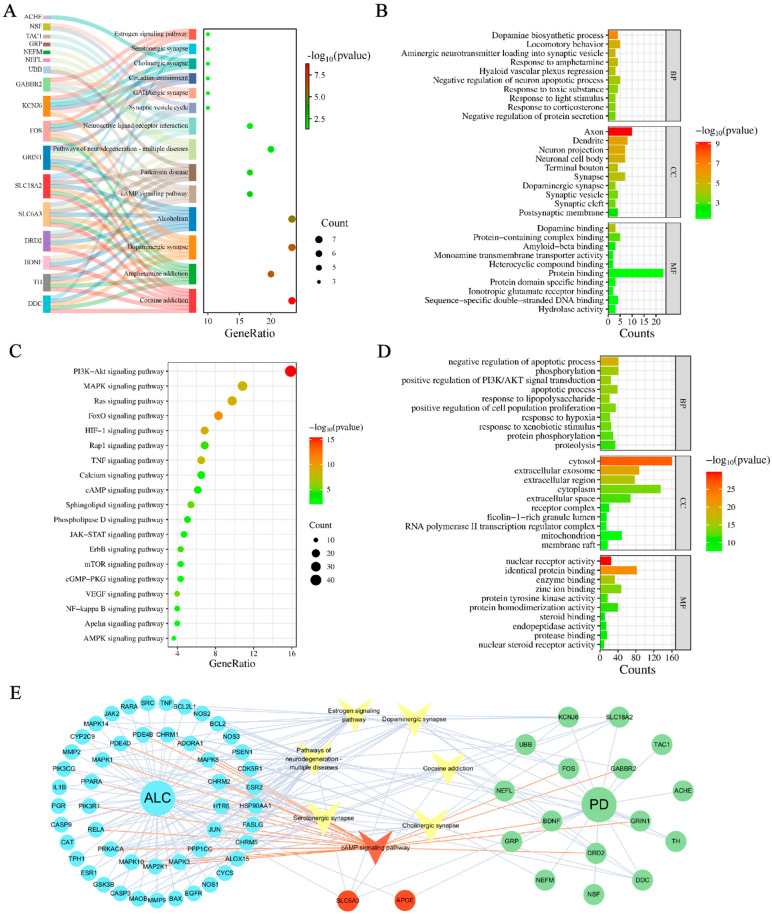
Exploration of key targets and pathways of ALC against PD. In the GO analysis, the top ten items with the highest significance among BP, CC, and MF are selected for presentation. (**A**) KEGG pathway enrichment analysis of PD hub genes. (**B**) GO functional enrichment analysis of PD hub genes. (**C**) KEGG pathway enrichment analysis of ALC targets. (**D**) GO functional enrichment analysis of ALC targets. (**E**) Target-pathway-disease interaction network. The ALC targets are blue, the PD hub genes are green, and the pathways are yellow.

KEGG analysis revealed that the pathogenesis of PD involves 14 pathways (Figure 2A), including the dopaminergic synapse and cAMP signaling pathway. GO analysis indicated that PD hub genes primarily affect several biological processes (BPs), including dopamine biosynthesis, locomotory behavior, and negative regulation of neuron apoptotic processes. The molecular functions (MFs) of these genes include dopamine binding and amyloid-beta binding, and their cellular components (CCs) are primarily localized in axons, dendrites, neuron projections, and neuronal cell bodies (Figure 2B).

KEGG analysis showed that the pharmacological mechanisms of ALC involve 19 signaling pathways, including PI3K-Akt, MAPK, and cAMP signaling pathways (Figure 2C). Notably, within the cAMP signaling pathway, ALC was enriched at 17 targets, including *PRKACA*, a key upstream regulator of this pathway. GO analysis revealed that ALC primarily affects biological processes such as the negative regulation of apoptotic processes, phosphorylation, and apoptotic processes. Its molecular functions encompass protein tyrosine kinase activity and nuclear receptor activity, with cellular localization concentrated in the cytoplasm (Figure 2D).

By intersecting ALC targets and pathways with PD-related elements, key targets associated with the anti-PD effects of ALC were identified, including *SLC6A3* and *APOE*. Key pathways included estrogen signaling, cAMP signaling, cholinergic synapses, neurodegeneration pathways—multiple diseases, serotonergic synapses, cocaine addiction, and dopaminergic synapses. These results suggest that ALC may exert its anti-PD effects through the cAMP signaling cascade. The final target–pathway–disease network is shown in Figure 2E.

### 2.3. Molecular Docking

**Figure 3:**
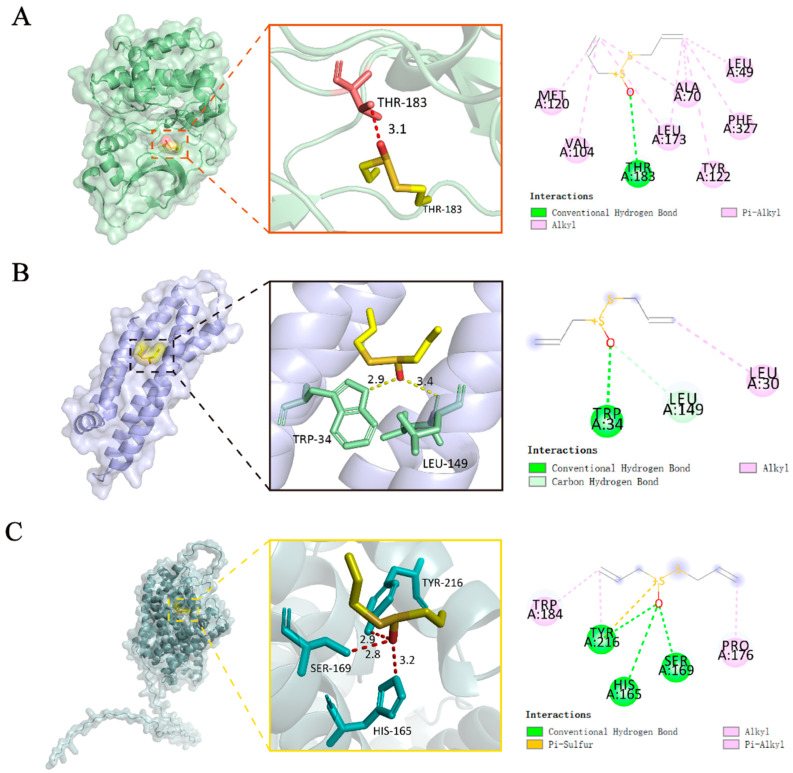
Molecular docking results. (**A**) Docking of ALC with PKA. (**B**) Docking of ALC with DAT. (**C**) Docking of ALC with APOE.

Molecular docking was performed to predict the binding affinity and mode of action of ALC with its targets. The docking of ALC with DAT (Q01959), PKA (3POO), and APOE (8AX8) was carried out using CB-DOCK2 (Figure 3). The dopamine transporter (DAT), encoded by *SLC6A3*, is a marker of dopaminergic neurodegeneration. APOE consists of a class of proteins closely related to the conversion and metabolism of lipoproteins. The docking results showed that the binding energies of ALC with PKA, DAT, and APOE were −4.5, −3.5, and −3.4 kcal/mol, respectively, and all formed hydrogen bonds, indicating that ALC exhibits good binding affinity with these targets in their natural state. We will further investigate the DAT and PKA targets in subsequent experiments. Since APOE was not enriched in relevant pathways in the KEGG analysis (Figure 2A) and scored lower among the 28 core targets, it will not be validated in subsequent experiments.

### 2.4. Neuroprotective Effect of ALC in 6-Hydroxydopamine (6-OHDA)-Induced Injury

**Figure 4:**
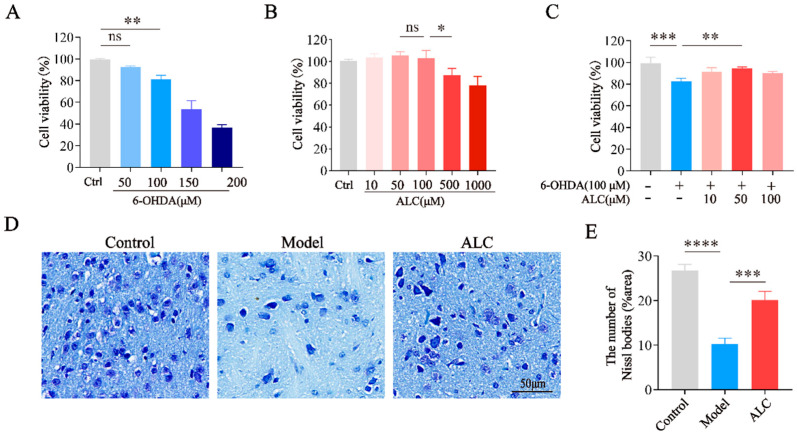
In vitro and in vivo neuroprotective effects of ALC. (**A**) Cytotoxicity of 6-OHDA at varying concentrations in SH-SY5Y cells. (**B**) Cytotoxicity of ALC at varying concentrations in SH-SY5Y cells. (**C**) Protective effects of ALC on 6-OHDA-induced toxicity in SH-SY5Y cells. (**D**) Representative images of Nissl staining. Scale bar = 50 μm. (**E**) Quantitative analysis of Nissl bodies (*n* = 3). ns represents not significant, * *p* < 0.05, ** *p* < 0.01, *** *p* < 0.001, **** *p* < 0.0001.

6-OHDA is a selective catecholaminergic neurotoxin that induces Parkinsonian pathology by generating reactive oxygen species and inhibiting mitochondrial complex I. The cell viability of SH-SY5Y cells treated with different concentrations of 6-OHDA and ALC was assessed using the CCK-8 assay. To determine the optimal 6-OHDA concentration for establishing the in vitro PD model, we first tested a gradient of 6-OHDA concentrations (0–200 μM) and observed their effects on cell viability. As shown in Figure 4A, exposure to 6-OHDA reduced cell viability in a concentration-dependent manner, with 100 μM of 6-OHDA inducing a significant decrease (*p* < 0.01) that was sufficient to mimic dopaminergic neuron damage in PD without causing excessive cell death. Consequently, this concentration was selected to establish the in vitro PD model. At a concentration of 500 μM, ALC caused a slight decrease in cell viability, and at 1 mM, the cells were significantly damaged (Figure 4B). SH-SY5Y cells were cotreated with 100 μM of 6-OHDA and different concentrations of ALC (10, 50, and 100 μM) for 24 h. The results showed that compared to the group treated only with 6-OHDA, cells treated with ALC exhibited less damage. Moreover, 50 μM of ALC significantly rescued viability in 6-OHDA-lesioned SH-SY5Y cells (Figure 4C). This indicates that ALC exerts a neuroprotective effect on the 6-OHDA-induced in vitro PD model.

Nissl staining was performed to observe the survival of striatal neurons in PD mice (Figure 4D). Nissl staining showed that, compared to the control group, the Nissl bodies in the model group exhibited deformed structures and a significant reduction in density. Compared to the model group, ALC treatment significantly increased Nissl body counts (Figure 4E) and restored their morphological organization and distribution to levels comparable with the control group. This suggests that ALC exerts a neuroprotective effect in PD mice.

### 2.5. ALC Improves Motor Dysfunction and Dopaminergic Neuron Loss in PD Mice

**Figure 5:**
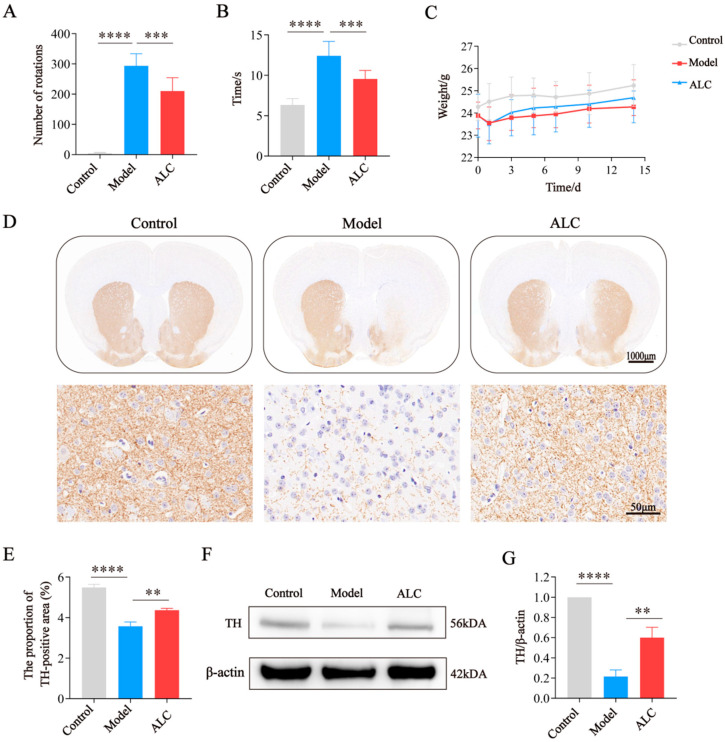
Effect of ALC on motor impairments and dopaminergic neurons in PD mice (*n* = 8). (**A**) APO-induced rotation test, (**B**) Pole climbing test, (**C**) Body weight changes, (**D**) Schematic diagram of TH immunohistochemistry. Scale bar = 1000 μm. (**E**) Quantitative analysis of TH immunohistochemistry (*n* = 3). (**F**) Western blot results of TH protein. (**G**) Quantitative analysis of TH protein expression (*n* = 3). ** *p* < 0.01, *** *p* < 0.001, **** *p* < 0.0001.

The apomorphine (APO)-induced rotation test was used to assess dopaminergic neuronal damage (Figure 5A). Compared to the model group, the ALC group exhibited a significant reduction in the number of rotations, suggesting that ALC alleviates dopaminergic neuronal damage and improves motor deficits caused by it. The Pole climbing test was used to evaluate the motor coordination of the mice (Figure 5B). Compared to the control group, the model group exhibited a significant increase in the time during the descent. In contrast, the ALC group spent less time during the descent, indicating that ALC improves motor coordination in PD mice. Changes in body weight during the experiment are shown in Figure 5C. After surgery, the mice lost weight rapidly, which slightly recovered following ALC treatment.

TH is a marker protein of dopaminergic neurons. Immunohistochemical analysis revealed that, compared to the control group, the expression levels of TH in the substantia nigra and striatum were significantly reduced in the model group (Figure 5D,E). Following ALC intervention, TH expression levels were increased, consistent with the WB results (Figure 5F,G). These suggest that ALC reduces dopaminergic neuronal damage and improves motor dysfunction.

### 2.6. ALC Inhibits Neuronal Apoptosis and Improves Neuroinflammation

**Figure 6:**
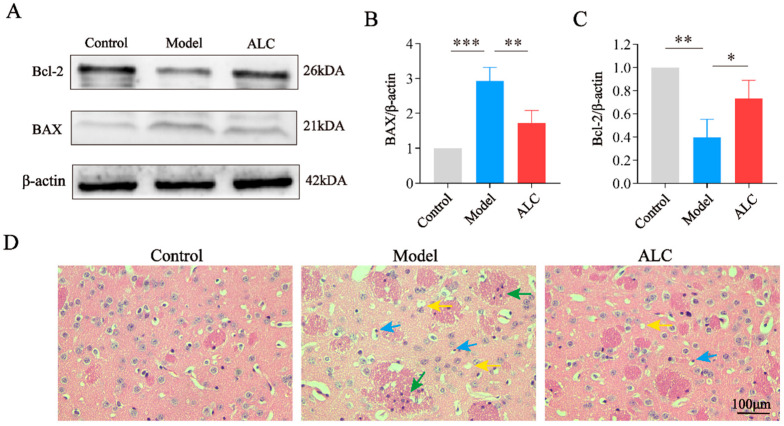
Effect of ALC on neuronal apoptosis in PD mice. (**A**) Western blot results of Bcl-2 and Bax. (**B**) BAX/β-actin (*n* = 3). (**C**) Bcl-2/β-actin (*n* = 3). (**D**) Representative H&E staining images. Scale bar = 100 μm. Blue arrow: karyopyknosis; yellow arrow: vacuole; green arrow: microglial nodule. * *p* < 0.05, ** *p* < 0.01, *** *p* < 0.001.

Western blot results revealed upregulated pro-apoptotic protein BAX and downregulated anti-apoptotic protein Bcl-2 expression in the model group versus controls. Following ALC intervention, BAX levels were downregulated while Bcl-2 was upregulated (Figure 6A–C). These results demonstrate ALC’s regulatory effect on apoptosis-associated proteins, thereby attenuating neuronal apoptosis.

H&E staining was performed to observe the pathological changes in the brain tissue of PD mice. H&E staining revealed normal brain tissue architecture with well-organized cellular morphology in the control group. In contrast, the model group exhibited pathological alterations, including nuclear pyknosis and vacuolation. Concomitantly, surrounding microglia demonstrated activation and proliferation, forming distinct microglial nodules. These findings suggested the occurrence of neuronal apoptosis, accompanied by a local immune response. Following treatment with ALC, these pathological changes were ameliorated to a certain extent, and tissue morphology more closely resembled that of the control group. These findings indicate that ALC attenuates neuroinflammation and cellular apoptosis (Figure 6D).

### 2.7. ALC Upregulates the PKA/p-CREB/BDNF Signaling Pathway and Restores DAT Expression

**Figure 7:**
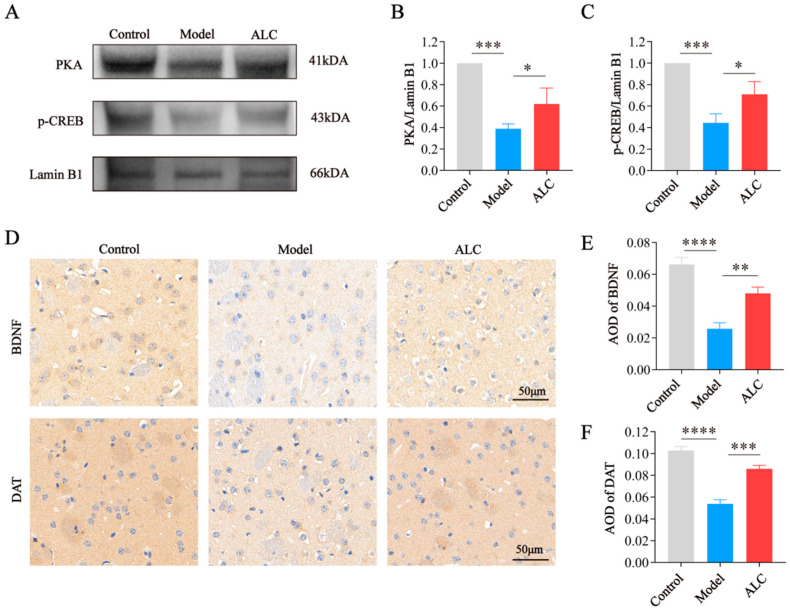
Effect of ALC on the PKA/p-CREB/BDNF signaling pathway. (**A**) Western blot results of PKA and p-CREB. (**B**) PKA/Lamin B1 (*n* = 3). (**C**) p-CREB/Lamin B1 (*n* = 3). (**D**) Representative immunohistochemistry images for DAT and BDNF. Scale bar = 50 μm. (**E**) Quantitative analysis of BDNF protein expression (*n* = 5). (**F**) Quantitative analysis of DAT protein expression (*n* = 5). * *p* < 0.05, ** *p* < 0.01, *** *p* < 0.001, **** *p* < 0.0001.

Western blot and immunohistochemistry results showed that, compared to the control group, the model group exhibited decreased levels of PKA, p-CREB (Figure 7A–C), BDNF, and DAT (Figure 7D–F). In contrast, the ALC group exhibited increased levels of PKA, p-CREB, BDNF, and DAT compared to the model group. The results indicate that ALC might promote neuronal survival by activating the PKA/p-CREB/BDNF signaling pathway and improve dopamine transport efficiency by restoring DAT protein expression, thereby exerting neuroprotective effects.

## 3. Discussion

PD is a common neurodegenerative disorder characterized primarily by tremors, rigidity, bradykinesia, and postural instability. ALC, the principal active component of garlic, exhibits notable antioxidant, anti-inflammatory, and neuroprotective properties; however, its underlying mechanisms in PD remain insufficiently studied. This study integrates bioinformatics, network pharmacology, and experimental validation to explore the effects and mechanisms of ALC against PD. Distinct from previous research, our work provides the first experimental validation of ALC in 6-OHDA-induced PD mice. Furthermore, we reveal, for the first time, that ALC exerts its neuroprotective effects by activating the PKA/p-CREB/BDNF signaling pathway and restoring DAT levels. Moreover, unlike conventional single-method pharmacological prediction strategies, this study integrates network pharmacology, bioinformatics, and in vitro/in vivo experimental validation. This multi-faceted methodology establishes a systematic research framework spanning target prediction to mechanistic verification, significantly enhancing the scientific rigor and credibility of mechanistic investigations into natural compounds.

First, we validated the neuroprotective effects of ALC against PD. The 6-OHDA induced PD model, recognized as a classic animal model for PD, was employed in this study, effectively representing the pathological features of PD patients [23]. The pole climbing test and APO-induced rotation test were utilized to assess the impact of ALC on the motor behavior of PD mice. The results indicated that the model group mice exhibited significantly increased climbing times and rotation counts, along with a marked reduction in TH expression levels in the substantia nigra and striatum, as well as a significant decrease in Nissl body count. This indicates that PD mice experienced motor deficits, accompanied by damage to dopaminergic neurons and dysfunction of neural cells. After ALC treatment, the climbing times and rotation counts significantly decreased, accompanied by a recovery in TH expression levels, as well as a notable increase in the count of Nissl bodies. This suggests that ALC can improve motor function in PD mice, mitigate the loss of dopaminergic neurons, and restore neuronal activity. These results demonstrate that ALC has a significant neuroprotective effect on PD mice, contributing to the alleviation of motor deficits.

Secondly, we explored the pathogenesis of PD and the pharmacological mechanisms of ALC. Studies indicate a significant correlation between apoptosis and the loss of dopaminergic neurons in PD; compared to the control group, the substantia nigra in PD patients exhibits marked apoptotic changes [24]. In the nervous system, the cAMP signaling pathway plays a crucial role in regulating various physiological functions, including neuronal survival, proliferation, and apoptosis. The downstream effector enzyme PKA phosphorylates various biological substrates, particularly CREB, a key transcription factor. CREB regulates the expression of genes associated with cell survival and proliferation, including BDNF. BDNF plays a critical role in neuroprotection, neural plasticity, and neuronal survival. It has been confirmed that upregulating the PKA/p-CREB/BDNF signaling pathway plays an important role in inhibiting neuronal apoptosis. Based on the analysis of five transcriptomic datasets from the substantia nigra of PD patients, we identified 28 PD hub genes, including TH, SLC6A3, and BDNF. GO-BP and KEGG enrichment analyses indicate that the pathogenesis of PD is closely related to apoptosis, and this biological process is likely influenced by the regulation of the classic PKA/p-CREB/BDNF pathway within the cAMP signaling pathway, aligning with previous studies.

As the main active component in garlic, ALC has been confirmed to inhibit neuronal apoptosis [25,26]. Concurrently, an early study has indicated that ALC can upregulate the activity of the PKA/p-CREB signaling pathway induced by 1,3-DCP in HepG2 cells [27]. Furthermore, another study has shown that ALC promotes the restoration of BDNF levels in the brains of rats with brain injury, demonstrating its neuroprotective effects [28]. These results suggest that ALC may play a role in the regulation of the PKA/p-CREB/BDNF signaling pathway. Based on network pharmacology, we identified 278 ALC targets, including PRKACA and SLC6A3. GO-BP and KEGG enrichment analyses indicate that ALC can influence apoptosis and is involved in the regulation of the cAMP signaling pathway. Integrating existing research and network predictions, we hypothesize that the PKA/p-CREB/BDNF pathway may constitute a key mechanism by which ALC exerts its anti-PD effects, where inhibition of apoptosis plays a crucial role.

To further validate the anti-apoptotic effects of ALC, we analyzed H&E staining and apoptosis-related proteins in mouse brain tissues. The results showed that the brain tissues in the model group exhibited typical apoptotic features such as nuclear condensation and vacuolization, with upregulation of the pro-apoptotic protein BAX and downregulation of the anti-apoptotic protein Bcl-2. In the ALC group, the cellular morphology and arrangement in brain tissues were similar to those in the control group, with a significant decrease in BAX protein expression and a significant increase in Bcl-2 protein expression. Additionally, in vitro experiments were conducted using the SH-SY5Y human neuroblastoma cell line, which is widely employed as a cellular model of PD due to its ability to imitate impaired dopaminergic neurons and its susceptibility to neurotoxins relevant to PD pathology [29]. Experiments demonstrated that 50 μM of ALC can effectively inhibit 6-OHDA-induced cell apoptosis. These results indicate that ALC possesses significant anti-apoptotic effects both in vivo and in vitro. Finally, we investigated the regulatory effects of ALC on the PKA/p-CREB/BDNF signaling pathway. As the transporter responsible for the reuptake of dopamine from the synaptic cleft to the presynaptic terminal, dysfunction of DAT is considered one of the significant mechanisms in the pathogenesis of PD. In PD patients, the loss of DAT signal intensity can be as high as 40–50% at the time of diagnosis [30]. Studies have shown that promoting the activation of the PKA/p-CREB/BDNF signaling pathway can aid in the functional recovery of DAT [31]. IHC and WB analyses revealed that ALC significantly upregulated the activity of the PKA/p-CREB/BDNF signaling pathway, leading to increased expression of BDNF and DAT. DAT is regulated not only by PKA/CREB but can also indirectly influence PKA activity through dopamine signaling, forming a dynamic feedback loop. We speculate that the upregulation of DAT may be related to the direct regulatory effect of the transcription factor CREB and the indirect effect of BDNF in enhancing neuronal survival. In molecular docking experiments, we observed that ALC exhibited good binding affinity to both target DAT and PKA. This further supports the possibility that ALC may exert its anti-PD effects by acting on these targets.

Microglia are immune cells in the central nervous system, and their activation is closely related to neuroinflammation, which is one of the significant factors leading to the death of dopaminergic neurons [32,33]. H&E staining indicated activation and proliferation of microglia in the model group, which were significantly reduced following ALC treatment, suggesting that ALC exerts anti-inflammatory effects in PD mice. In the KEGG analysis, the ALC targets were significantly enriched in inflammatory pathways such as the PI3K/AKT pathways. The PI3K/AKT signaling pathway is considered crucial in the central nervous system due to its involvement in physiological processes such as cell survival, autophagy, neurogenesis, proliferation, and differentiation of neurons, and synaptic plasticity. Many natural products exert protective effects on dopaminergic neurons and inhibit microglial activation by activating the PI3K/AKT pathway, thereby playing a positive role in the prevention and treatment of PD [34]. Meanwhile, ALC has been shown to activate the PI3K/AKT pathway to alleviate cellular damage [35]. We hypothesize that the anti-inflammatory effects of ALC may be related to the regulation of these pathways, but further research is still needed to verify this.

## 4. Materials and Methods

### 4.1. Prediction of ALC Targets and Acquisition of PD-Related Genes

ALC targets were collected from various databases, including SwissTargetPrediction [36], PubChem [37], SymMap [38], PharMapper [39], CTD [40], and STITCH [41]. Transcriptional data sets of the substantia nigra from PD patients were obtained from the GEO database [42] using the R package (4.5.1) GEOquery, including GSE7621, GSE20292, GSE20163, GSE20164, and GSE49036. Differentially expressed genes (log_2_FC > 1, *p* < 0.05) were identified from each dataset, which were then used for the screening of PD hub genes.

### 4.2. PPI Network Construction and Selection of PD Hub Genes

PPI analysis was conducted on the differentially expressed genes from each dataset. A PPI network for drug-disease common targets was constructed using the STRING [43] platform with a minimum interaction threshold of 0.4 for medium confidence, while default values were applied for other parameters. Subsequently, the tsv file was downloaded and imported into Cytoscape (v3.10.2) for visualization. Identify hub nodes based on the MCC algorithm. The MCC values for each node were calculated using the CytoHubba plugin in Cytoscape (v3.10.2). In this study, the top 10 genes ranked by MCC value were considered hub genes. The hub genes from the five datasets were merged to obtain the hub genes associated with PD.

### 4.3. Pathway and Functional Enrichment

The DAVID database [44] was utilized to perform GO functional and KEGG pathway enrichment analyses on the PD hub genes and ALC target genes, with results visualized using the online tool Srplot [45].

### 4.4. Visualization of Target-Pathway-Disease Interactions

Common targets and pathways between the ALC targets and PD hub genes were identified using Venny 2.1.0 [46]. A target-pathway-disease network was constructed using Cytoscape (v3.10.2).

### 4.5. Molecular Docking

The 3D structures and converted formats of ligand compounds were obtained from the PubChem database, while the 3D structures of receptor proteins were retrieved from the PDB database [47] or AlphaFold [48]. Molecular docking was performed using the online platform CB-DOCK2 [49]. A binding energy of less than 0 kcal/mol indicates that the ligand and the receptor can bind spontaneously. Two-dimensional interaction diagrams were generated using Discovery Studio 2019, and visualization was performed with PyMOL.

### 4.6. Reagents and Preparation

All reagents were purchased from Macklin (Shanghai, China), Beyotime (Shanghai, China), Sigma-Aldrich (Merck KGaA, Darmstadt, Germany), and Servicebio (Wuhan, China). Antibodies were obtained from Proteintech (Rosemont, IL, USA), Servicebio (Wuhan, China), Nature Biosciences (Beijing, China), and UpingBio (Hangzhou, China).

### 4.7. Cell Culture and Viability Assay

SH-SY5Y cells were cultured in a complete medium consisting of 10% FBS and 1% penicillin/streptomycin in high-glucose DMEM, in a humidified incubator at 37 °C with 5% CO_2_. The CCK-8 assay was used to assess the effects of 6-OHDA and ALC on the viability of SH-SY5Y cells to evaluate chemical toxicity or neuroprotective activity. SH-SY5Y cells (1 × 10^4^ cells/well) were seeded into 96-well plates and incubated for 24 h. ALC (A875877, Macklin, purity ≥ 95% by HPLC) was dissolved in DMSO and diluted with saline to achieve a final DMSO concentration of less than 1‰ (*V/V*). The cells were exposed to various concentrations of 6-OHDA (50, 100, 150, and 200 μM) and ALC (10, 50, 100, 500, and 1000 μM) for 24 h, followed by the addition of 10 μL of CCK-8 solution to each well for a 2 h incubation, and the optical density (OD) values were measured at 450 nm using a microplate reader. Cell viability was calculated using the following formula: Cell viability (%) = (OD_treatment_/OD_control_) × 100%.

### 4.8. Animals and Treatment

All experimental procedures were conducted in accordance with the guidelines of the Animal Research Committee of Guangxi University (protocol code No. GXU-2025-003). Ten-week-old male C57BL6 mice were purchased from SPF Biotechnology Co., Ltd. (Beijing, China) and were housed under conditions of 50 ± 10% relative humidity, 22 ± 2 °C, and a 12 h light/dark cycle, with ad libitum access to food and water. After one week of acclimatization, the mice underwent three days of pole climbing training. The mice were randomly divided into a control group and a surgical group. The modeling method and drug dosage were referenced from the existing literature [50,51]. Using a stereotaxic apparatus, 6-OHDA (3.3 μg/μL, 2 μL per site) was unidirectionally injected into the striatum of the surgical group mice at two coordinates. Coordinate 1: ML 0.3 mm, AP 0.2 mm, DV −3.0 mm; coordinate 2: ML 1.1 mm, AP 1.7 mm, DV −2.9 mm. The needle was retained for 5 min post-injection before being slowly withdrawn. One week later, the APO-induced rotation test was conducted, with > 100 rotations in 30 min considered a successful model. Finally, the mice were divided into three groups (*n* = 8): control group, model group, and ALC group. The control and model groups received daily intraperitoneal injections of saline, while the ALC group received 50 mg/kg of ALC via intraperitoneal injection for 14 consecutive days, during which the body weight of the mice was recorded. After behavioral testing, the mice were euthanized, and brain tissues were collected.

### 4.9. APO-Induced Rotation Experiment

Following intraperitoneal injection of 0.1 mg/kg of APO, the mice were placed in a 50 × 50 cm open square arena. The number of rotations was recorded for 30 min once the mice began to rotate.

### 4.10. Pole Climbing Test

Mice were placed at the top of a 50 cm long rough wooden pole, which was covered with thin gauze, and the time taken for the mice to move from the top to the bottom was recorded. This process was measured three times to obtain an average value.

### 4.11. Nissl Staining and H&E Staining

The brain tissues were fixed in 4% paraformaldehyde, followed by dehydration, clearing, and paraffin embedding. Coronal sections of the embedded brain tissues were cut at a thickness of 20 μm and stained with Nissl staining solution and H&E staining, followed by dehydration, clearing, and mounting. Finally, the sections were observed and photographed under a microscope (Zeiss, Oberkochen, Germany). The number of Nissl bodies per field of view was quantified using ImageJ software (v1.54p).

### 4.12. Western Blot

Proteins were extracted from SH-SY5Y cells and the substantia nigra striatum using RIPA lysis buffer (RIPA: PMSF: phosphatase inhibitor = 100:1:1). Protein concentration was determined using a BCA protein assay kit (Beyotime), and proteins were separated by SDS-PAGE according to their molecular weights before being transferred to a PVDF membrane (300 mA for 1.5 h). The membrane was blocked with a rapid blocking solution for 1 h and then incubated overnight at 4 °C with rabbit anti-mouse primary antibodies against TH (1:5000, Proteintech), β-actin (1:5000, Proteintech), Bax (1:1000, UpingBio), Bcl-2 (1:1000, Proteintech), Lamin B1 (1:5000, Nature Biosciences), PKA (1:2000, UpingBio), and p-CREB (1:1000, Nature Biosciences). Subsequently, the membrane was incubated with a secondary antibody (1:5000, Servicebio) for 2 h. After applying ECL, the membrane was exposed and photographed using a gel imaging system (5200, Shanghai Tanon Life Science Co., Ltd., Shanghai, China), followed by analysis with ImageJ software (v1.54p).

### 4.13. Immunohistochemistry

Brain tissue was fixed in 4% paraformaldehyde and subjected to antigen retrieval using sodium citrate buffer. The tissue was then permeabilized with 0.25% Triton X-100 at room temperature and incubated overnight at 4 °C with anti-tyrosine hydroxylase antibody (1:500). The sections were washed with Tris-buffered saline and then incubated at room temperature for 1 h with a horseradish peroxidase-conjugated secondary antibody (1:200), followed by staining with 3,3′-diaminobenzidine and washing. The sections were scanned using a microscope, and the relevant parameters were analyzed with ImageJ software (v1.54p) to determine the average optical density (AOD) or proportion of positive areas.

### 4.14. Statistical Analysis

Western blot data were normalized using control group values as reference baselines to eliminate potential batch effects. Specifically, values for each indicator (model group, ALC group) were divided by their corresponding control group values, resulting in normalized values of 1.0 for all control groups. All data are presented as mean ± SD from ≥3 independent experiments. Statistical analyses were performed using GraphPad Prism 8. One-way ANOVA was employed to assess intergroup differences, with *p* < 0.05 considered statistically significant.

## 5. Conclusions

ALC ameliorates motor deficits and neuropathology in PD mice by activating the PKA/p-CREB/BDNF/DAT signaling pathway and inhibiting neuronal apoptosis. These findings provide novel mechanistic evidence supporting ALC’s neuroprotective effects in PD.

## Data Availability

The data presented are available from the corresponding author upon request.
